# Grazing-incidence synchrotron radiation diffraction studies on irradiated Ce-doped and pristine Y-stabilized ZrO_2_ at the Rossendorf beamline

**DOI:** 10.1107/S1600577524000304

**Published:** 2024-02-16

**Authors:** Volodymyr Svitlyk, Luiza Braga Ferreira dos Santos, Jonas Niessen, Sara Gilson, Julien Marquardt, Stefan Findeisen, Selina Richter, Shavkat Akhmadaliev, Nina Huittinen, Christoph Hennig

**Affiliations:** a Helmholtz-Zentrum Dresden-Rossendorf (HZDR), Institute of Resource Ecology, Dresden, Germany; b Rossendorf Beamline (BM20), European Synchrotron Radiation Facility, Grenoble, France; c RWTH Aachen University, Institute of Mineral Engineering, Aachen, Germany; d Goethe-University Frankfurt, Institute of Geosciences, Frankfurt, Germany; e Helmholtz-Zentrum Dresden-Rossendorf, Mechanical Engineering, Dresden, Germany; f Helmholtz-Zentrum Dresden-Rossendorf, Ion Beam Center, Dresden, Germany; g Freie Universität Berlin, Institute of Chemistry and Biochemistry, Berlin, Germany; RIKEN SPring-8 Center, Japan

**Keywords:** yttria-stabilized zirconia, irradiation, synchrotron radiation, grazing incidence diffraction, microstrain

## Abstract

In this work, Ce-doped yttria-stabilized zirconia (YSZ) and pure YSZ phases were subjected to irradiation with 14 MeV Au ions. Irradiation studies were performed to simulate long-term structural and microstructural damage due to self-irradiation in YSZ phases hosting alpha-active radioactive species. It was found that both the Ce-doped YSZ and the YSZ phases had a reasonable tolerance to irradiation at high ion fluences and the bulk crystallinity was well preserved. Nevertheless, local microstrain increased in all compounds under study after irradiation, with the Ce-doped phases being less affected than pure YSZ. Doping with cerium ions increased the microstructural stability of YSZ phases through a possible reduction in the mobility of oxygen atoms, which limits the formation of structural defects. Doping of YSZ with tetravalent actinide elements is expected to have a similar effect. Thus, YSZ phases are promising for the safe long-term storage of radioactive elements. Using synchrotron radiation diffraction, measurements of the thin irradiated layers of the Ce-YSZ and YSZ samples were performed in grazing incidence (GI) mode. A corresponding module for measurements in GI mode was developed at the Rossendorf Beamline and relevant technical details for sample alignment and data collection are also presented.

## Abstract 2

Irradiated Ce-doped yttria-stabilized zirconia (YSZ) and pure YSZ phases were characterized using grazing incidence (GI) synchrotron radiation diffraction. A corresponding module for measurements in GI mode was developed at ROBL and relevant technical details of sample alignment and data collection are presented.

## 1. Introduction

Spent nuclear fuel (SNF) from nuclear reactors is disposed of directly or after reprocessing. In the latter case, immobilization of specific waste streams, such as minor actinide-containing waste, in durable crystalline host matrices would enhance the long-term safety of these installations. Phases based on yttria-stabilized zirconia (YSZ) are promising candidates for this purpose. Indeed, tetravalent actinides can be easily incorporated into the crystalline YSZ matrices, and corresponding solubility ranges were found to depend on the symmetry of the parent phases (Svitlyk *et al.*, 2022*a* ▸). Specifically, a more symmetrical arrangement of oxygen atoms in ZrO_8_ polyhedra was found to result in a higher intake of tetravalent actinide elements. Doped YSZ-based phases were also found to exhibit excellent stability at elevated temperatures with no discharge of incorporated guest tetravalent atoms (Svitlyk *et al.*, 2022*b* ▸). Similarly, application of external pressure did not induce changes in the chemical compositions of doped cubic and tetragonal YSZ phases, despite the phase transition induced in the tetragonal YSZ modification at 9 GPa (Svitlyk *et al.*, 2022*b* ▸). This shows the excellent affinity of incorporated tetravalent guest atoms with the host YSZ matrices under extreme temperature and pressure conditions. The latter is an important factor to consider when evaluating phases as potential hosts for radioactive elements for long-term storage of nuclear waste. In fact, undergoing radioactive α-decay leads to the formation of alpha particles (He^2+^) and finally to the formation of He gas (Wiss *et al.*, 2014 ▸). Formed He bubbles trapped inside the host crystalline matrix lead to an increase in local pressure which may even reach 10 GPa (van Brutzel & Chartier, 2015 ▸).

In addition to the pressure effect, alpha particles and high-energy recoil nuclei generated during the self-irradiation process may alter the microstructure of the host crystalline material. In compounds containing radioactive species, this damage may be manifested through the generation of point defects, swelling, increased strain, reduced crystallinity or may even lead to complete amorphization (Kato *et al.*, 2009 ▸; Weber *et al.*, 1986 ▸; Foltyn *et al.*, 1985 ▸; Booth *et al.*, 2007 ▸; Headley *et al.*, 1981 ▸). Induced microstructural changes may subsequently influence mechanical, chemical and physical properties of corresponding phases and, therefore, effects of radiation damage must be studied for materials intended to host radioactive species for long periods of time. The effect of self-irradiation can be simulated by bombarding phases of interest with heavy ions and the corresponding technique is known as ion beam irradiation or ion implantation. By bombarding materials with energetic (kiloelectronvolts to megaelectronvolts range) ions of He (to simulate alpha particles) or ions of heavy elements (typically to simulate the recoil of daughter products), such as gold or noble gases, defects and microstructural damage can be created in corresponding crystal structures. The possibility to change the fluence of generated ion beams allows for controlled and accelerated experiments that can reveal mechanisms and kinetics of radiation damage in different materials (Sickafus *et al.*, 1999 ▸; Shu *et al.*, 2020 ▸; Yan *et al.*, 2015 ▸; Lenz *et al.*, 2019 ▸; Leys *et al.*, 2022 ▸).

In this paper, we report grazing incidence (GI) diffraction studies on irradiated pellets of Ce-doped and pure YSZ. The cerium (IV) cations are used as surrogates for the tetravalent actinides because they have similar ionic radii and physicochemical properties (Feuchter *et al.*, 2019 ▸; Lopez *et al.*, 2005 ▸; Sweet *et al.*, 2017 ▸). Irradiation experiments were performed at the Ion Beam Center (HZDR, Dresden, Germany) with two fluences to simulate the different scenarios of self-irradiation during waste storage in underground repositories.

GI synchrotron radiation diffraction experiments were performed at the Rossendorf Beamline (ROBL BM20, ESRF, Grenoble, France). It is a powerful technique which allows for the investigation of structural changes in materials induced by irradiation. By employing GI geometry, it is possible to selectively probe thin irradiated layers near the surface of samples, where the X-ray penetration depth is small at low scattering angles (Lenz *et al.*, 2019 ▸; Dutta *et al.*, 2018 ▸; Wang *et al.*, 2023 ▸; Simeone *et al.*, 2002 ▸). A corresponding analysis of irradiation-induced microstructural changes in the Ce-YSZ and YSZ phases was performed. Although conventional GI diffraction studies can be successfully performed using laboratory X-ray sources [Simeone *et al.* (2011 ▸, 2013 ▸) and references therein], utilization of synchrotron radiation may provide additional technical and scientific advantages. Firstly, orders-of-magnitude-higher flux available at synchrotron facilities compared with laboratory sources results in significant reduction in data collection time. This allows various *in situ* GI studies to be performed, for example as a function of temperature (Perez–Taborda *et al.*, 2018 ▸; Foran *et al.*, 1998 ▸), to follow chemical reactions (Blair *et al.*, 2023 ▸; Carino *et al.*, 2003 ▸) or even to perform GI tomographic scans (Tsai *et al.*, 2021 ▸). Also, the possibility to fine-tune the energy of the incoming synchrotron radiation allows us to perform anomalous GI studies where the contribution of a specific element of interest to the diffraction signal can be enhanced or reduced (Renaud *et al.*, 2009 ▸; Lee *et al.*, 2005 ▸). Reported here are the first studies at the ROBL performed in GI mode on samples treated *ex situ* (irradiated). This opens new possibilities for more complex *in situ* studies mentioned above, in particular on radioactive samples. We also provide a technical description of the experimental setup designed and built at ROBL in order to perform diffraction experiments in GI mode. Corresponding sample alignment and measurement procedures are also presented.

## 2. Experimental

### 2.1. Sample synthesis

Three Ce-doped YSZ samples were synthesized via co-precipitation in order to obtain sample targets for the current study. Both cerium and yttrium concentrations in the samples were varied to obtain the following compositions: Ce_0.18_Y_0.15_Zr_0.67_O_1.93_, Ce_0.18_Y_0.20_Zr_0.62_O_1.90_ and Ce_0.58_Y_0.15_Zr_0.27_O_1.93_. For the synthesis, ZrOCl_2_·8H_2_O (Sigma-Aldrich) was dissolved in 0.01 *M* HCl under magnetic stirring. Then an appropriate amount of a 2.76 *M* Ce-stock solution prepared by dissolving CeCl_3_·7H_2_O (Alfa Aesar) in 0.01 *M* HCl was added to the Zr^4+^-containing solution. Yttrium was introduced from 2.699 *M* YCl_3_·6H_2_O stock solution (Alfa Aesar). Precipitation of the hydrous zirconia precursors occurred after dropwise addition of 12.5% NH_4_OH (Sigma–Aldrich) to the Ce/Y/Zr solutions. The precipitates were washed several times with doubly deionized water (Milli-Q grade). After the last washing step, the samples were dried at 80°C for 24 h. The dry powders were then re-suspended in iso­propyl alcohol, and 5% mass of polyethyl­ene glycol (PEG) was added to the suspension to assist grain agglomeration. The slurries were mortared twice in a ball mill for 2 min. The samples were placed in a fume hood for 48 h to evaporate all the remaining iso­propyl alcohol. The dry solids were pressed to pellets under a uniaxial pressure of ∼400 MPa for 1 min. Sample calcination was performed at 1500°C for 2 h. In total, six pellets of each composition (two for each fluence) were produced with the weight of each ceramic varying between 2 and 3 g. Representative pellet densities are given in Table 1 ▸. Densities of sintered pellets were determined geometrically (Francis, 2016 ▸). The thickness and diameter of each pellet were measured multiple times and these values were averaged to improve accuracy.

A commercial YSZ sample with 15 mol% Y (Y_0.15_Zr_0.85_O_1.93_) was obtained from Tosoh. The powder was isostaticaly pressed in a rubber mold under an uniaxial pressure of ∼400 MPa for a duration of 1 min, followed by a gradual pressure release. Produced YSZ pellets were heated at a rate of 5°C min^−1^ and sintered at 1450°C for 2 h. The sintered pellets had a density of 5.8 g cm^−3^, determined using the Archimedean method.

### 2.2. Sample irradiation

Ce-doped YSZ and YSZ samples were irradiated at the Ion Beam Center (HZDR, Dresden, Germany). To prepare for the irradiation, the pellets were carefully polished. First, the pellet surfaces were smoothed using CarbiMet 600 [P1200] silicon carbide paper and then polished on a polishing table with 1 µm diamond paste until a mirror-like condition was achieved. The polished targets were then mounted on silicon wafers using copper tape with good heat conductivity. One half of each pellet was masked with Al foil in order to protect the pristine side from the ion beam. Irradiation was conducted using 14 MeV Au^4+^ ions at two fluences of 1 × 10^14^ ions cm^−2^ (referred to as F1 in the text) and 1 × 10^15^ ions cm^−2^ (F2). The samples were cooled in a liquid-nitro­gen-refrigerated cryostat during irradiation to minimize sample surface heating by the ion beam.

Theoretical estimations and the penetration depth of the incident ions were calculated using the Monte Carlo simulation code *SRIM* [*Stopping Range of Ions in Matter* (Ziegler *et al.*, 2010 ▸)] in combination with experimentally determined pellet densities and crystallographic formulae.

### 2.3. SEM analysis

Microstructural characterization was conducted utilizing scanning electron microscopy (SEM) and energy dispersive spectroscopy (EDS) (FESEM Gemini 500 by Zeiss, Oberkochen, Germany; EDS detector X-Max80 by Oxford Instruments, Abingdon, Oxfordshire, UK). The samples were not coated due to subsequent surface sensitive measurements. Utilization of an acceleration voltage of 1 kV yielded high-quality secondary electron images without inducing surface charging. However, for acquiring backscattered images, 15 kV acceleration voltage was used in variable-pressure mode. This reduced vacuum condition facilitated charge equalization at the surface through interactions with gas molecules.

### 2.4. GI synchrotron radiation diffraction setup and experimental procedure

**Figure 1:**
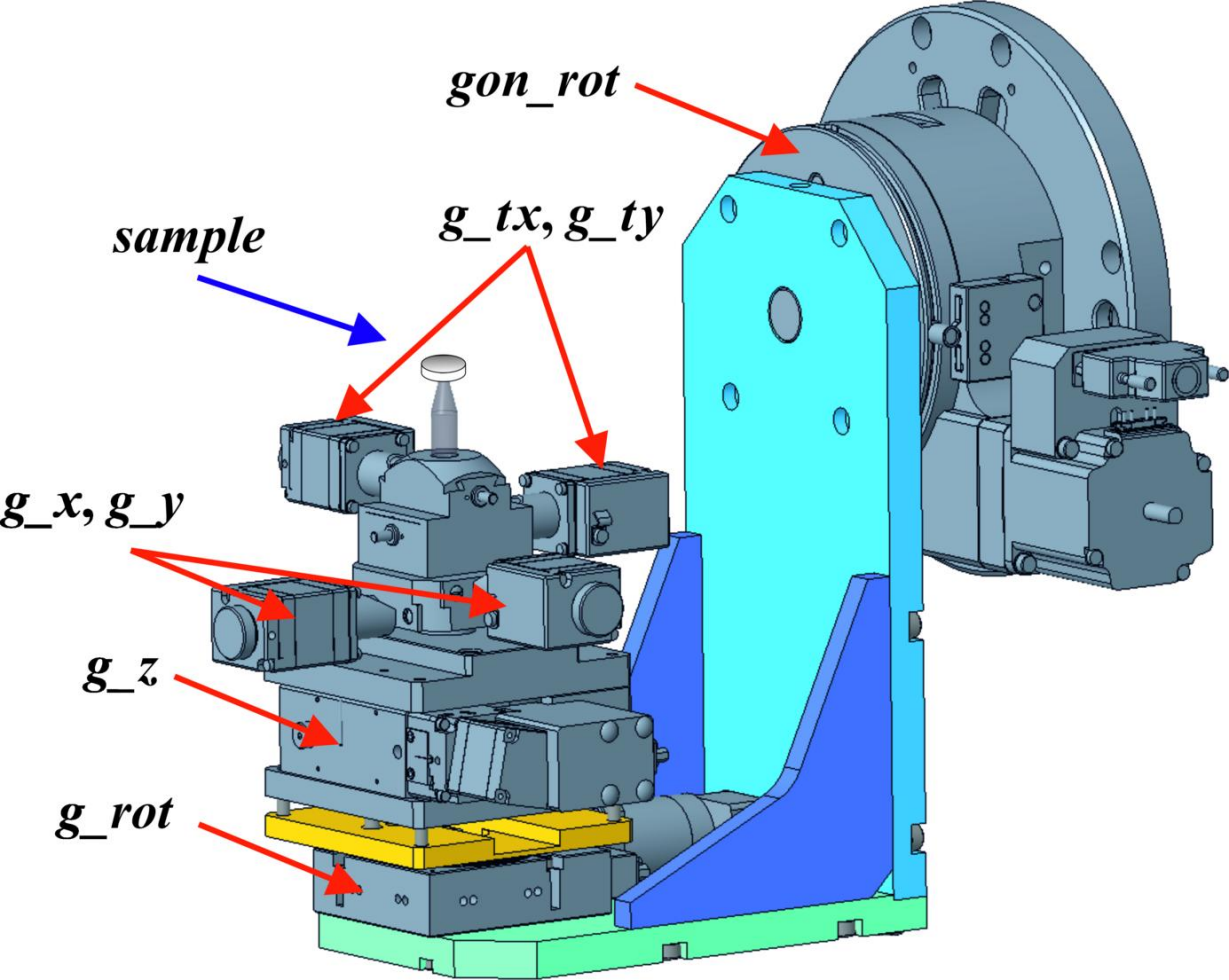
Technical drawing of the GI module of the ROBL, illustrating the degrees of freedom available for sample alignment; the *gon_rot* rotation axis is used to change the grazing incidence angle α during data collection.

Diffraction experiments in grazing mode were performed at the Rossendorf Beamline (ROBL BM20, ESRF, Grenoble, France). For this, a special setup was designed, machined and subsequently assembled in-house. A corresponding technical drawing is shown in Fig. 1 ▸.

A flat sample to be measured (*e.g.* a dense pellet) is fixed on a sample holder (marked as *sample* in Fig. 1 ▸). The pellet is subsequently aligned with respect to the incoming beam. For this, motorized vertical translation (*g_z*), perpendicular horizontal translations (*g_x* and *g_y*) and perpendicular tilting (*g_tx* and *g_ty*) arcs are used. Initially, the pellet is positioned in the beam with a vertical scan *g_z* using a half-cut of the X-ray beam. Then it is placed in the rotation center of the *g_rot* goniometer using two perpendicular horizontal translations *g_x* and *g_y*. Finally, the pellet is aligned parallel to the beam with *g_tx* and *g_ty* tilting translations using, again, half-cut scans. The GI module is designed in a way that the position of the aligned pellet coincides with the position of the *gon_rot* rotation axis center used to change the incidence angle α. All alignment is performed at α = 0 and for data collection a predefined set of incidence angles is measured. For this GI module, translational accuracy is 1 µm and rotational accuracy is better than 0.01°.

**Figure 2:**
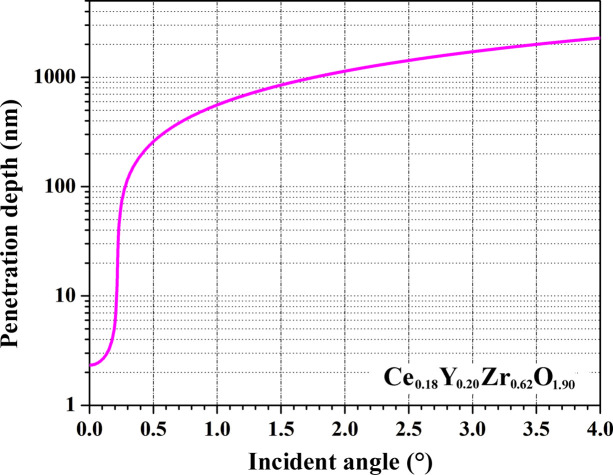
Penetration depth of the synchrotron radiation with an energy of 11.1 keV into Ce_0.18_Y_0.20_Zr_0.62_O_1.90_ (ρ = 4.86 g cm^−3^) as a function of grazing angle α simulated with the *GIXA* suite.

Initially, a critical scattering angle (β) for the studied phase at a specific photon energy has to be calculated. Subsequent data collection is performed at discreet grazing incidence angles (α), which are larger than the critical angle β. The choice of α is dictated by the desired X-ray penetration depth which is phase- (chemical composition and density) and energy-dependent and should be calculated. For the Ce-doped YSZ and YSZ samples studied here, the critical angles (β) and penetration depths as a function of α were calculated using the *GIXA* package (https://gixa.ati.tuwien.ac.at/; Ingerle *et al.*, 2016 ▸) which is based on X-ray scattering data published by Henke *et al.* (1993 ▸) (Fig. 2 ▸, calculations for the Ce_0.18_Y_0.20_Zr_0.62_O_1.90_ sample are shown as an example). During data collection the sample can be oscillated by 360° in order to improve diffraction statistics from samples with a coarse-grain microstructure. During oscillation, the planarity of the sample with respect to the incidence beam is assured by the half-cut scans performed previously along the two orthogonal *x* and *y* directions (Fig. 1 ▸). The Ce-doped YSZ and YSZ samples studied here were collected with angular oscillations of ±20° which allowed us to collect pristine and irradiated parts separately from the half-irradiated sides of the measured pellets.

The GI module was mounted on the XRD2 multipurpose diffractometer of ROBL (Scheinost *et al.*, 2021 ▸). Calibration of the GI diffraction setup was performed with a standard NIST 660c LaB_6_ powder. For this, a small quantity of LaB_6_ was deposited on a glass slide, and then ethanol was dripped onto the powder to achieve a uniform dispersion. As a result, a compact thin film was formed. The sample obtained was collected at incidence (α) angles of 1°, 1.5°, 2°, 3°, 4°, 5°, 6°, 8°, 10°, 12° and 14°. For the measurements, the energy of the incoming synchrotron beam was set to 11.1 keV and the beam was focalized and slitted down to a 0.4 mm (horizontal) and 0.03 mm (vertical) size. Data were recorded on a Pilatus 2M detector and reduced to 1D powder patterns with the *PyFAI* module (Kieffer & Karkoulis, 2013 ▸) as implemented into the *BUBBLE* suite (Dyadkin *et al.*, 2016 ▸).

**Figure 3:**
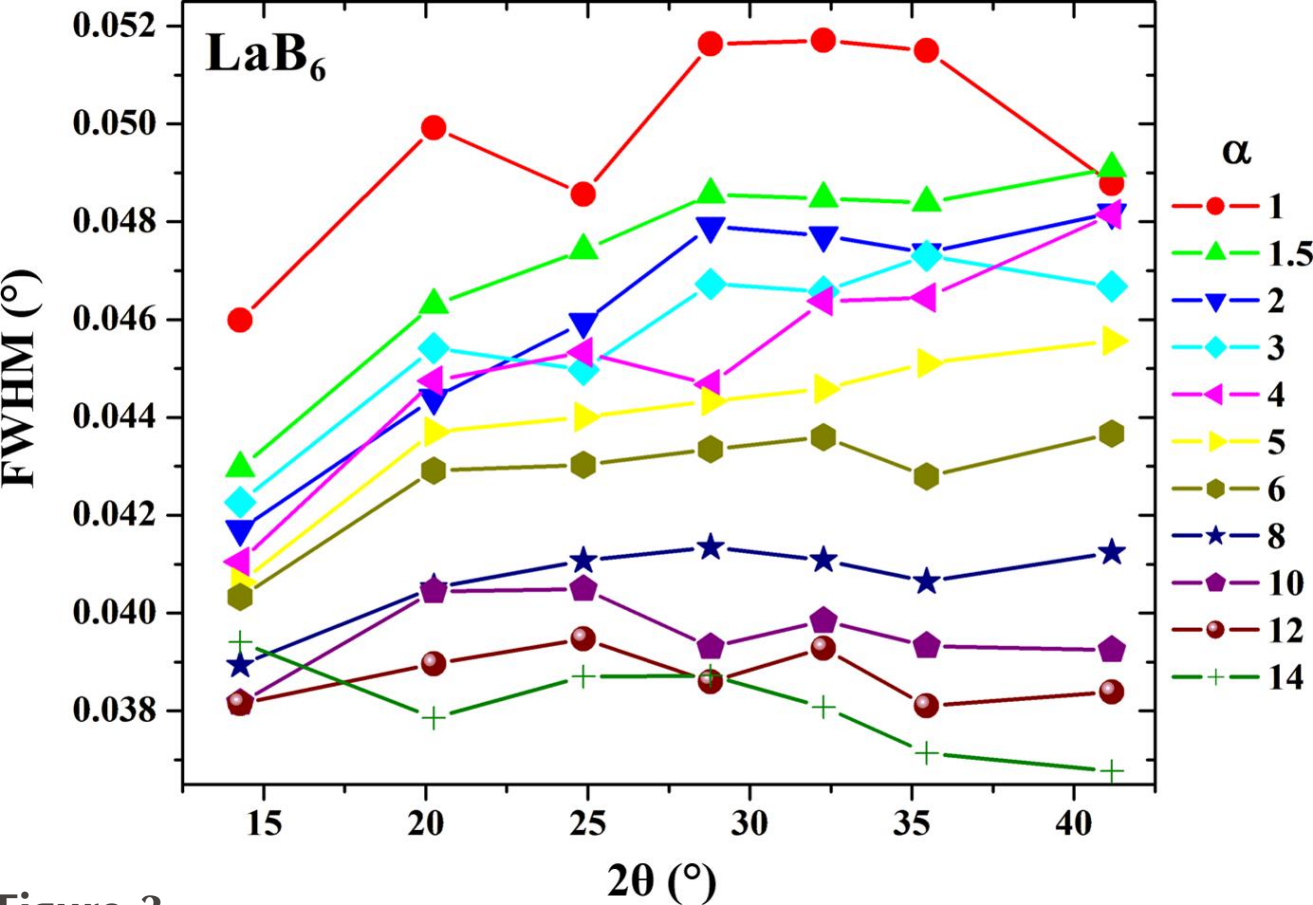
FWHM of the LaB_6_ diffraction profile as a function of scattering 2θ and α incidence angles.

Diffraction data collected in GI mode on the LaB_6_ standard sample allows us to follow the effective footprint of the incident synchrotron beam. In grazing mode, the beam footprint contributes significantly towards peak-broadening and this effect is expected to be the largest at the lowest incidence angles. Indeed, systematic analysis of the full width at half-maximum (FWHM) for the diffraction data collected on the LaB_6_ standard shows a steady decrease in peak width with an increase in the incidence angle α (Fig. 3 ▸). The FWHM–2θ dependencies obtained can be used to subtract effective experimental contributions from the beam footprint to peak-broadening for diffraction data collected on both pristine and irradiated samples. This correction was implemented here by introducing instrumental resolution functions (IRFs) during Rietveld refinement in the *FullProf* program (Rodriguez-Carvajal, 2001 ▸) for the corresponding incidence angles (Fig. 3 ▸). The use of IRFs also allows the subtraction of all the effective instrumental errors within the observed profile. Therefore, no additional theoretical calculations are needed to model these contributions. All diffraction data for the Ce-doped YSZ and YSZ samples studied here were treated using the Le Bail + IRF method.

## 3. Results and discussion

**Figure 4:**
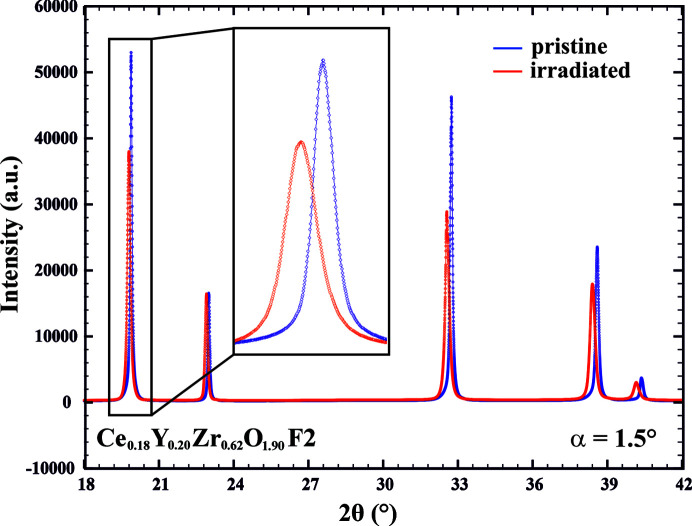
Diffraction patterns of pristine (blue line) and irradiated (red line) parts of the Ce_0.18_Y_0.20_Zr_0.62_O_1.90_ – F2 sample.

For all the Ce-doped YSZ and non-doped YSZ samples, a comparison of the GI XRD data from the unirradiated and irradiated parts shows that all the samples resisted irradiation with heavy ions quite well. Specifically, Bragg peaks still remain well defined (Fig. 4 ▸, Ce_0.18_Y_0.20_Zr_0.62_O_1.90_ – F2 is shown as an example), indicating that the long-range 3D order is preserved for these materials even after irradiation at higher fluences. Similar microstructural resistance to irradiation at different fluences and with other heavy ions (*i.e.* Xe^2+^ or I^+^) was observed for both stabilized and non-stabilized zirconia (Sickafus *et al.*, 1999 ▸). Nevertheless, pure monoclinic zirconia or hafnia were found to exhibit a crystalline-to-crystalline phase transition on irradiation (Sickafus *et al.*, 1999 ▸; Benyagoub, 2005 ▸). In contrast, bombardment with large Cs^+^ ions was reported to induce amorphization in cubic zirconia (Wang *et al.*, 2000 ▸) and this behavior is dictated by a large size-mismatch between Zr^4+^ of the zirconia matrix and the implantation ions. Indeed, the ionic radius of Cs^+^ in coordination number VIII is equal to 1.74 Å and these ions are twice as large as Zr^4+^ with a radius of 0.84 Å (Shannon, 1976 ▸). For comparison, the I^+^ species are less than 1 Å in size (Ozeki & Saito, 2004 ▸) and the Au^4+^ ions are smaller than the Zr^4+^ ions [*R*_ION_(Au^4+^) ≃ 0.7 Å].

**Figure 5:**
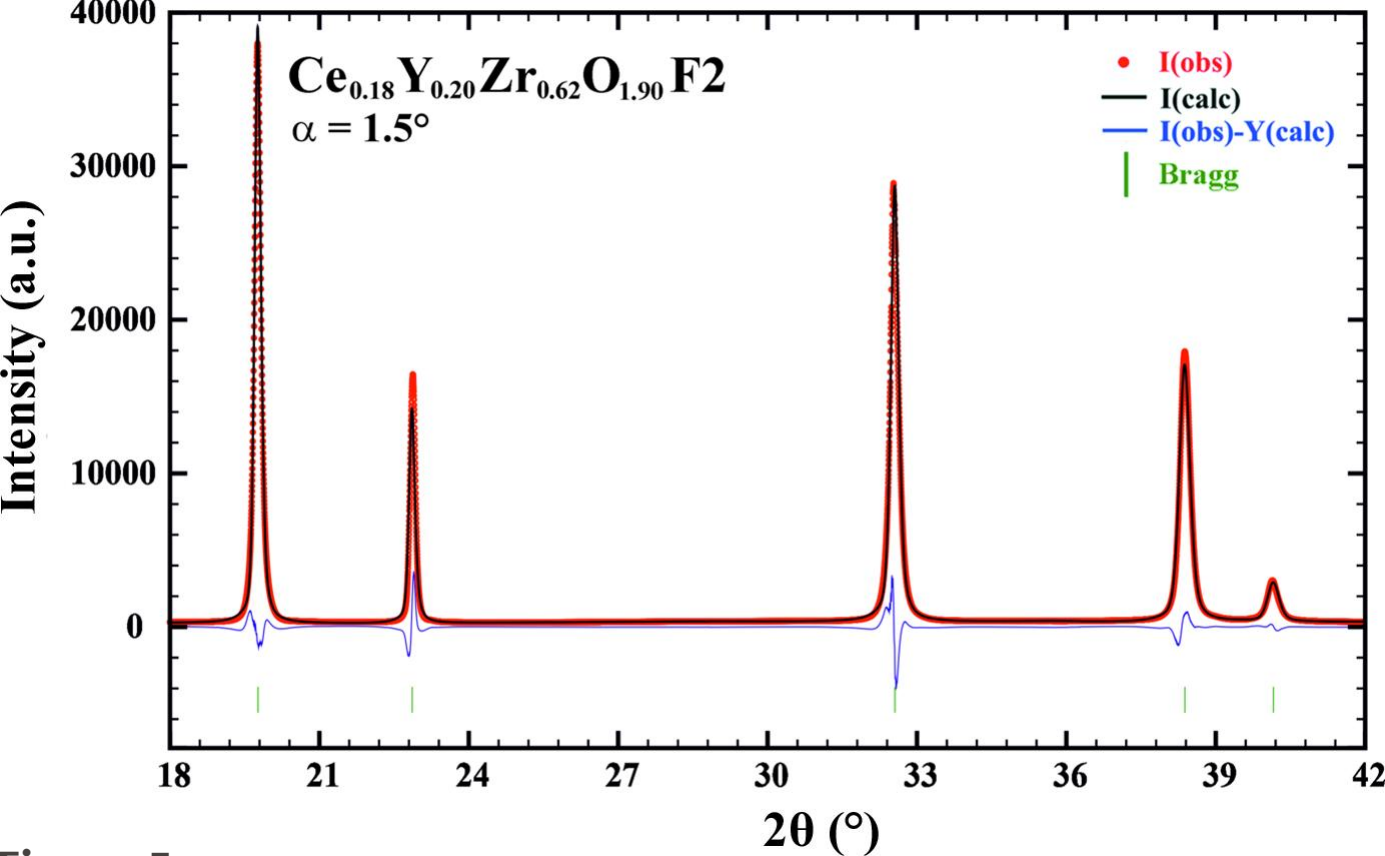
Full-profile Rietveld refinement of the irradiated Ce_0.18_Y_0.20_Zr_0.62_O_1.90_ – F2 sample in Le Bail mode (*R*_P_ = 7.8%). Experimental data are represented by red points: *I*(obs); the calculated profile is shown as a continuous black line: *I*(calc); the continuous blue line is the difference between the experimental data and the calculated profile: *I*(obs) − *I*(calc); the green vertical bars are the Bragg positions. Results of refinement for other samples in the Ce-YSZ and YSZ series are presented in Figs. S1–S6 of the supporting information.

However, a closer look (Fig. 4 ▸, inset) shows that a certain broadening of the peaks can be observed. This implies that irradiation induced some microstructural changes: a reduction in domain size, an increase in strain or a combination of both. Displacement of peak positions can be observed as well, which may indicate the presence of induced strains or a change in unit-cell volume (swelling). It can be concluded that the strain effect is present since the Bragg peaks of the irradiated phase (Fig. 4 ▸, red profile) are shifted towards smaller Bragg angles compared with the parent peaks of the pristine phase (Fig. 4 ▸, blue profile) in a non-systematic way. Indeed, full microstructural analysis performed with the *FullProf* package in Le Bail mode (Fig. 5 ▸, Ce_0.18_Y_0.20_Zr_0.62_O_1.90_ – F2 phase shown as an example) revealed that irradiation significantly increases internal strain in all the studied YSZ-based materials. Specifically, Ce-doped YSZ phases exhibit an increase in microstrain by a factor of 1.6–1.9 (Table 2 ▸) and the induced damage is similar for the two employed fluences (F1 and F2). This indicates excellent microstructural stability of these phases against irradiation and they are expected to tolerate even higher fluences without significant changes in the microstructure.

On the contrary, the non-doped YSZ sample was more significantly affected by the external irradiation with heavy ions. Specifically, microstrain of YSZ was increased by a factor of 4.0 for the F2 fluence. This indicates that doping with large tetravalent Ce^4+^ ions has a stabilizing effect on the YSZ phases against induced irradiation damage. Indeed, within the Ce-doped YSZ series, samples with the highest Ce content exhibit the lowest relative increase in induced microstrain after irradiation at both F1 and F2 fluences (Table 2 ▸). Another distinct difference between the Ce-doped YSZ and non-doped YSZ phases is the effective penetration depth of heavy ions used for the irradiation studies. This depth can be estimated from GI diffraction data by tracking the appearance of signal from the bulk on increase in the incidence angle α, and comparing it with theoretically calculated penetration depth of the employed radiation (*e.g.* synchrotron in the current study). As concluded from the experimental GI data and corresponding theoretical calculations (Fig. 2 ▸), the effective ion penetration depth for the Ce-YSZ samples is situated around the 1.5–2 µm range. However, GI diffraction data for the YSZ sample indicate the penetration depth of heavy ions is ∼0.8 µm. This implies that the non-doped YSZ sample has a higher stopping power than the Ce-YSZ analogs. Subsequently, a higher energy is deposited by irradiating ions per unit volume of the crystalline lattice of YSZ with a resulting increase in induced strain. A higher ion penetration depth for the Ce-doped phases may be explained by the creation of voids in the parent YSZ matrix by introduction of larger Ce^4+^ ions [*R*_ION_(Ce^4+^) = 0.97 Å versus *R*_ION_(Zr^4+^) = 0.84 Å] with an overall expansion in the unit cell (Table 3 ▸). Indeed, non-doped YSZ is denser (ρ = 5.8 g cm^−3^) than its Ce-doped analogs (ρ = 4.86–5.42 g cm^−3^, Table 1 ▸). Theoretical *SRIM* calculations of the penetration depth of 14 MeV Au ions into the pellets studied agree with the experimentally observed trend. Namely, Au ions were found to penetrate approximately 2.5 µm and 1.9 µm within the Ce-YSZ and the YSZ pellets, respectively (Figs. S7 and S8 of the supporting information). This systematic positive shift with respect to the estimations based on scattering power (Fig. 2 ▸) originates from the convention which defines penetration depth of the incoming radiation (*e.g.* synchrotron). It is defined as the depth at which the intensity is reduced to 1/*e* (∼37%) of the original intensity at the surface. Thus, about 1/3 of the photons of the intense synchrotron beam are still available at this depth and propagate further into the material. Therefore, effective penetration depths, as obtained experimentally from GI experiments and corresponding theoretical estimations based on scattering power (Fig. 2 ▸), are systematically lower than those obtained theoretically with *SRIM*.

Materials based on zirconia are known to exhibit high mobility of oxygen atoms at elevated temperatures and, therefore, these systems have enormous potential as solid oxide fuel cells (Zakaria *et al.*, 2020 ▸; Vinchhi *et al.*, 2023 ▸; Maiti *et al.*, 2022 ▸). A similar mobility may also be expected to occur on irradiation due to induced thermal spikes (Miotello & Kelly, 1997 ▸; Vineyard, 1976 ▸; Benyagoub, 2005 ▸) which are characterized by localized and rapid (approximately pico­second scale) increases in temperature within the material. Indeed, irradiation is known to induce loss of oxygen in ZrO_2_-based materials with a resulting stoichiometry on the oxygen site even reaching ∼1.65 (Zhang *et al.*, 2010 ▸; Edmondson *et al.*, 2011 ▸). Certainly, the formation of oxygen vacancies induces lattice distortions (Raza *et al.*, 2016 ▸) which may result in the generation of residual microstrains (Edmondson *et al.*, 2011 ▸). Introduction of Ce^4+^ ions into the YSZ matrix would certainly alter the oxygen conductivity of the YSZ phases during the irradiation-induced thermal spikes and would influence the resulting stoichiometry. Indeed, it was found that the bulk conductivity in Ce-doped YSZ phases exhibits U-shape behavior as a function of Ce content (Ananthapadmanabhan *et al.*, 1990 ▸; Yang *et al.*, 2011 ▸). Initially, the conductivity of YSZ samples decreases on introduction of Ce ions. This response was explained by the formation of defects and increased scattering of oxygen ions. The electrical conductivity reaches a minimum at the equimolar Zr:Ce ratio, where the probability of oxygen ions encountering dissimilar cations is at a maximum. As a result, the oxygen pathway is severely distorted and conductivity reaches a minimum. Further increase in Ce content results in improved oxygen mobility due a combination of two effects: a more homogeneous chemical environment and an increase in lattice volume. Correspondingly, bulk conductivity increases steadily and reaches a second maximum at the Ce-rich part of the phase diagram. This behavior correlates well with the microstructural response under irradiation in the Ce-YSZ series observed here. Namely, phases rich in Ce feature smaller irradiation-induced increase in microstrain (Table 2 ▸) and this can be related to the reduced oxygen mobility with a resulting preservation of local structure. A similar stabilization effect is also expected to occur on doping of YSZ with large tetravalent actinide elements. Specifically, oxygen mobility would decrease and the resulting microstructural stability against internal irradiation would increase.

The presence of oxygen vacancies was also shown to influence the radiation tolerance of zirconia-based materials. In particular, creation of these vacancies in ZrO_2_ by introduction of trivalent yttrium ions increased the radiation tolerance due to highly effective dynamic annealing arising from the interaction of defects produced by radiation with structural vacancies, as found by molecular dynamics simulations (Devanathan & Weber, 2008 ▸). Specifically, the presence of oxygen vacancies in YSZ provides radiation-generated interstitial oxygen atoms with a quick one hop path to be annihilated at a vacancy located in the adjacent position. The resulting recombination on the anion sublattice triggers corresponding cation defect recombination. Although there are variations in oxygen content within the Ce-YSZ and YSZ samples studied here, doping with Ce^4+^ ions is found to fully predominate this effect.

**Figure 6:**
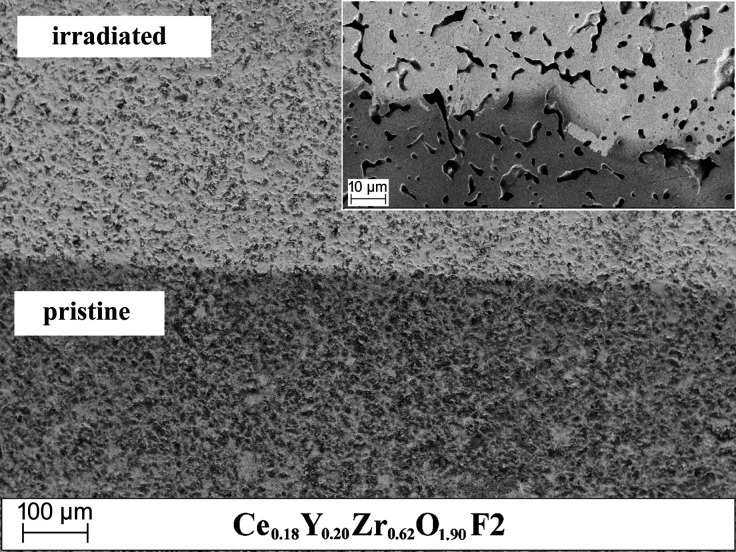
SEM images of the pristine (bottom) and irradiated (top, F2) Ce_0.18_Y_0.20_Zr_0.62_O_1.90_ sample surface. The pristine part was preserved by covering the sample with Al foil. The inset shows the magnified boundary between the pristine and irradiated parts.

Full-profile microstructural analysis revealed no size-induced peak-broadening both for the Ce-doped YSZ and for the non-doped YSZ samples. Indeed, it was observed that irradiation with heavy ions could even induce an increase in the grain size of nanocrystalline zirconia (Zhang *et al.*, 2010 ▸; Edmondson *et al.*, 2011 ▸). In this material, about a four-fold increase in grain size was triggered by irradiation and this irradiation-induced grain growth can be described by a thermal spike model (Kaoumi *et al.*, 2008 ▸). Here grain boundaries are directly affected by thermal spikes and this mechanism is also valid at low temperatures where grain growth remains unaffected by irradiation temperature. According to this model, the movement of grain boundaries is driven by atomic jumps that take place within the thermal spikes, and this movement is influenced by the local curvature of the grain boundaries. Observed preservation of domain size for the Ce-doped YSZ and non-doped YSZ phases on irradiation, as seen from GI diffraction data, also agrees with the experimental SEM analysis performed on these samples (Fig. 6 ▸, Ce_0.18_Y_0.20_Zr_0.62_O_1.90_ – F2, pristine and irradiated parts of the sample are shown as an example). Indeed, no apparent size-related effects like grain segregation or swelling are visible for irradiated samples.

Examination of the unit-cell volume, as obtained from full-profile Rietveld Le Bail analysis (Figs. 5 ▸ and S1–S6 of the supporting information), revealed an irradiation-induced volume increase for the Ce-doped YSZ and non-doped YSZ series. Samples irradiated with F1 fluence feature an increase in unit-cell volume on the order of 1.0% (Table 3 ▸). Samples irradiated with F2 fluences exhibit slightly larger swelling of ∼1.3%. Thus, in contrast to microstrain, irradiation at higher fluences has an effect on the induced swelling, although it is rather limited.

Interestingly, close examination of the unit-cell volume of pristine samples (Table 3 ▸) reveals a small difference between analogous F1 and F2 series of samples. However, the relative change in unit-cell volume between the respective non-irradiated samples is insignificant compared with the swelling induced by irradiation – 0.2% for Ce_0.18_Y_0.15_Zr_0.67_O_1.93_ and Ce_0.18_Y_0.20_Zr_0.62_O_1.90_, and 0.1% for Ce_0.58_Y_0.15_Zr_0.27_O_1.93_. This minor difference can stem from inevitable centering uncertainties (convolution of sample planarity and beam size) and from the preparation procedure. Specifically, independent pressing of different pellets also may result in slight local dispersion in the densities of obtained samples with related small changes in lattice parameters and/or strains. Indeed, deviations in internal strains were also observed for the same F1–F2 pairs of pristine samples (Table 2 ▸).

## 4. Conclusions

In addition to the already documented excellent stability under extreme conditions of temperature or pressure, YSZ-based compounds also feature remarkable resistance against irradiation. This characterizes the YSZ phases as robust materials that would retain their chemical, physical and mechanical properties over time in underground storage conditions. Integrity of phases hosting radioactive species is crucial for the safety of corresponding storage facilities. YSZ phases are, therefore, promising candidates as hosts for major or minor tetravalent actinides. Introduction of large Ce^4+^ ions into YSZ was found to be beneficial for the microstructural stability of these phases. This behavior is likely to be dictated by the associated reduction in oxygen mobility and resulting reduced damage to the crystal lattice. Introduction of large tetravalent actinides is expected to trigger a similar mechanism of microstructural preservation. Solubility properties of the YSZ phases have yet to be investigated in order to be able to assess their stability with respect to leaching from groundwater. Such studies are planned as the next step in the evaluation of the YSZ-based materials for nuclear applications.

The complex microstructural characterization presented in this paper relied on a combination of an intense and small synchrotron beam and a custom-designed GI diffraction setup. The GI module used for this study is available on BM20 ROBL for similar studies where only the top thin layers of flat and dense samples need to be characterized. These can be irradiated or other materials with chemically or physically modified surfaces, including radioactive samples. Time-resolved *in situ* studies on heating, anomalous diffraction measurements or tracking of chemical reactions in grazing mode are also possible. Access to the HZDR BM20 beamline may be granted for external users through standard ESRF proposal.

## Supplementary Material

Rietveld refinement profiles of the Ce-YSZ and YSZ samples; SRIM calculations for penetration depth of 14 MeV Au ions into the Ce-doped and non-doped YSZ phases. DOI: 10.1107/S1600577524000304/yi5147sup1.pdf

## Figures and Tables

**Table 1:** Pellet compositions and experimental densities

Composition	Thickness (mm)	Diameter (mm)	Volume (cm^3^)	Mass (g)	Density (g cm^−3^)
Ce_0.18_Y_0.15_Zr_0.67_O_1.93_	4.54	9.36	0.31	1.63	5.23
Ce_0.18_Y_0.20_Zr_0.62_O_1.90_	4.61	9.67	0.34	1.65	4.86
Ce_0.58_Y_0.15_Zr_0.27_O_1.93_	5.26	10.57	0.46	2.51	5.42

**Table 2:** Observed microstrain in the Ce-YSZ and YSZ pristine and irradiated samples at fluences F1 = 1 × 10^14^ ions cm^−2^ and F2 = 1 × 10^15^ ions cm^−2^

Sample	Strain, pristine (%)	Strain, irradiated (%)	Factor
Ce_0.18_Y_0.15_Zr_0.67_O_1.93_ – F1	17	32	1.9
Ce_0.18_Y_0.20_Zr_0.62_O_1.90_ – F1	20	33	1.7
Ce_0.58_Y_0.15_Zr_0.27_O_1.93_ – F1	43	70	1.6
Ce_0.18_Y_0.15_Zr_0.67_O_1.93_ – F2	24	42	1.8
Ce_0.18_Y_0.20_Zr_0.62_O_1.90_ – F2	41	73	1.8
Ce_0.58_Y_0.15_Zr_0.27_O_1.93_ – F2	34	54	1.6
YSZ (Y_0.15_Zr_0.85_O_1.93_) – F2	14	56	4.0

**Table 3:** Irradiation-induced changes in the volume of the unit cells in Ce-doped YSZ and non-doped YSZ samples

Sample	Unit-cell volume, pristine (Å^3^)	Unit cell volume, irradiated (Å^3^)	Relative increase (%)
Ce_0.18_Y_0.15_Zr_0.67_O_1.93_ – F1	139.801 (1)	141.239 (1)	1.0
Ce_0.18_Y_0.20_Zr_0.62_O_1.90_ – F1	140.061 (1)	141.330 (1)	0.9
Ce_0.58_Y_0.15_Zr_0.27_O_1.93_ – F1	150.347 (1)	151.823 (1)	1.0
Ce_0.18_Y_0.15_Zr_0.67_O_1.93_ – F2	140.097 (1)	141.763 (1)	1.2
Ce_0.18_Y_0.20_Zr_0.62_O_1.90_ – F2	139.712 (1)	141.640 (1)	1.4
Ce_0.58_Y_0.15_Zr_0.27_O_1.93_ – F2	150.507 (1)	152.392 (1)	1.3
YSZ (Y_0.15_Zr_0.85_O_1.93_) – F2	135.566 (1)	136.917 (1)	1.0
