# Supplementary material for: Grazing-incidence synchrotron radiation diffraction studies on irradiated Ce-doped and pristine Y-stabilized ZrO2 at the Rossendorf beamline
Source: J Synchrotron Radiat. 2024 Feb 16;31(Pt 2):355–62. doi: 10.1107/S1600577524000304 (PMC10914159; doi:10.1107/S1600577524000304)
Supplement: Supplementary file 1 [file s-31-00355-sup1.pdf]

JOURNAL OF SYNCHROTRON RADIATION

Volume 31 (2024)

Supporting information for article:

Grazing-incidence synchrotron radiation diffraction studies on irradiated Ce-doped and pristine Y-stabilized ZrO2 at the Rossendorf beamline

Volodymyr Svitlyk, Luiza Braga Ferreira dos Santos, Jonas Niessen, Sara Gilson, Julien Marquardt, Stefan Findeisen, Selina Richter, Shavkat Akhmadaliev, Nina Huittinen and Christoph Hennig

Grazing incidence synchrotron radiation diffraction studies on irradiated Ce-doped and pristine Y-stabilized ZrO_2 at the Rossendorf Beamline

Volodymyr Svitlyk[1,2*], Luiza Braga Ferreira dos Santos[1], Jonas Niessen[3], Sara Gilson[1], Julien Marquardt[4], Stefan Findeisen[5], Selina Richter[1], Shavkat Akhmadaliev[6], Nina Huittinen[1,7], Christoph Hennig[1,2*]

*svitlyk@esrf.fr, hennig@esrf.fr

[1]Helmholtz-Zentrum Dresden-Rossendorf, Institute of Resource Ecology, Dresden, Germany

[2]Rossendorf Beamline (BM20), European Synchrotron Radiation Facility, Grenoble, France

[3]RWTH Aachen University, Institute of Mineral Engineering, Aachen, Germany

[4]Goethe-University Frankfurt, Institute of Geosciences, Frankfurt, Germany

[5]Helmholtz-Zentrum Dresden-Rossendorf, Mechanical Engineering, Dresden, Germany

[6]Helmholtz-Zentrum Dresden-Rossendorf, Ion Beam Center, Dresden, Germany

[7]Freie Universität Berlin, Institute of Chemistry and Biochemistry, Berlin, Germany

Supporting information

Rietveld refinement profiles of the Ce-YSZ and YSZ samples

In the following figures experimental data are represented by red points; the calculated profile is shown as a continuous black line; the continuous blue line is the difference between the experimental data and the calculated profile; green vertical bars are the Bragg positions. We note that asymmetry in peak shape was observed to varying degrees in the experimental data for Ce-YSZ and YSZ. It likely originates from a non-perfect planarity of pressed pellets compared to the highly focused and parallel incoming beam.

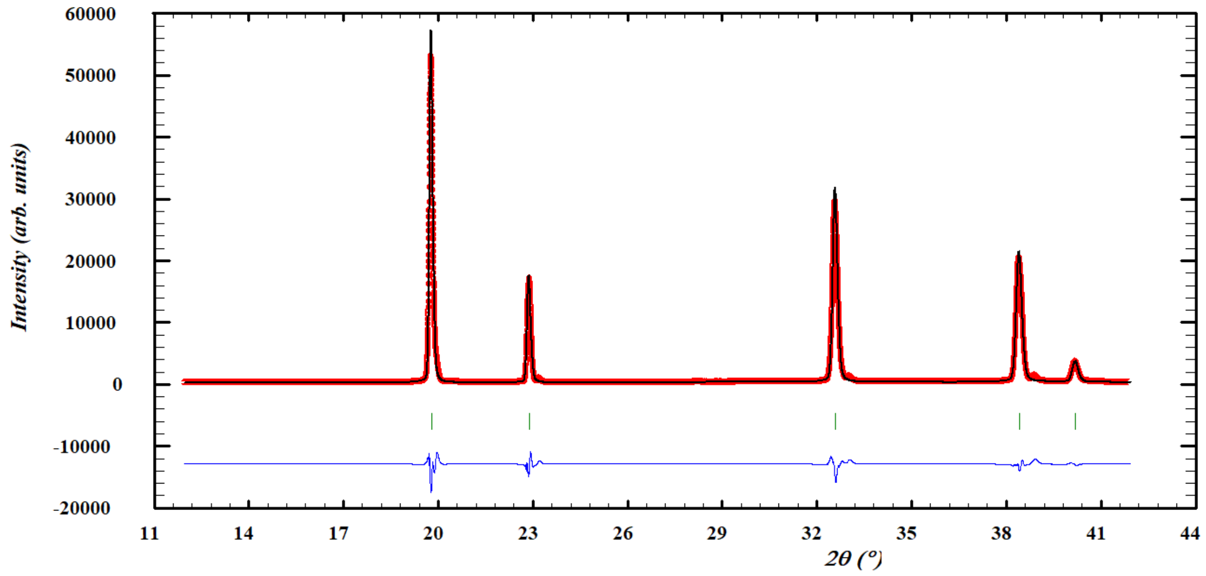

Fig. 1S. Full-profile Rietveld refinement of the irradiated $Ce_{0.18}Y_{0.15}Zr_{0.67}O_{1.93}$ – F1 sample in a Le Bail mode (R_P = 6.6%).

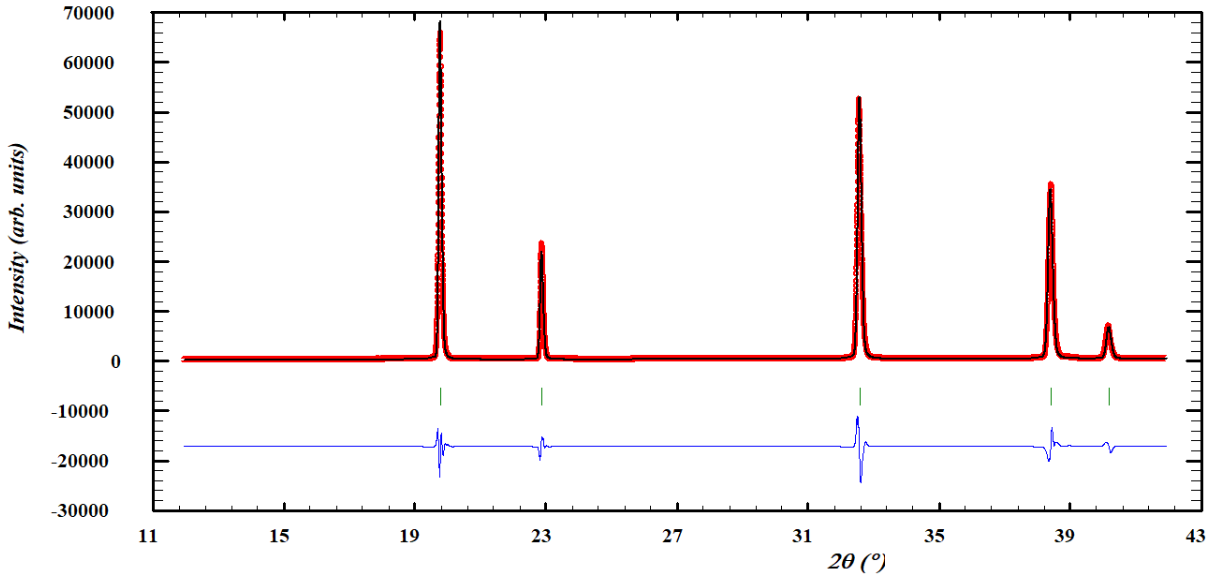

Fig. 2S. Full-profile Rietveld refinement of the irradiated $Ce_{0.18}Y_{0.20}Zr_{0.62}O_{1.90}$ – F1 sample in a Le Bail mode (R_P = 8.6%).

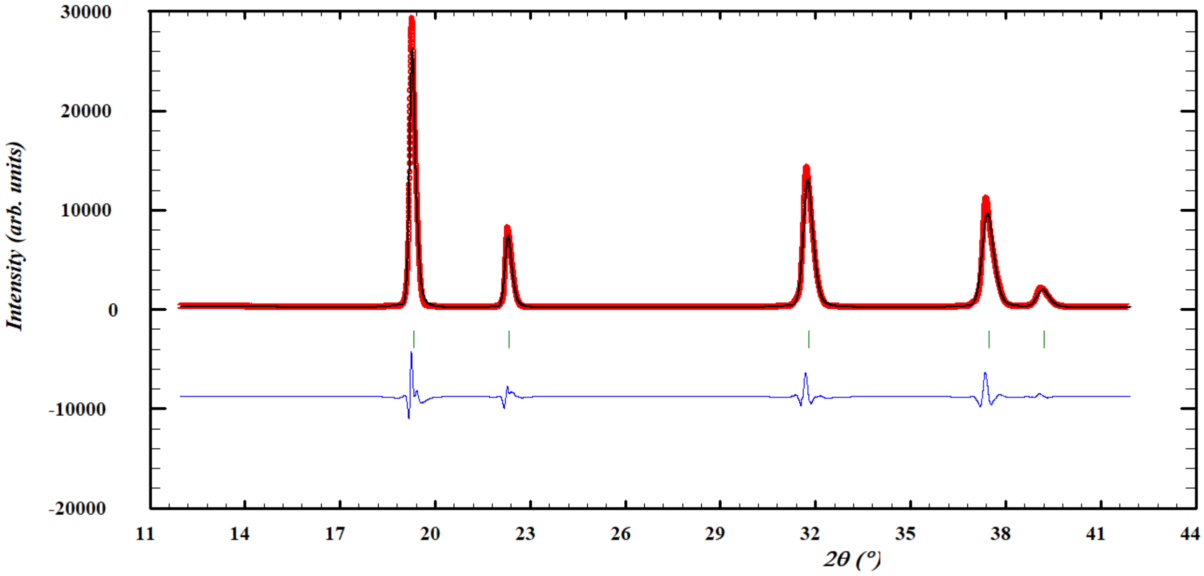

Fig. 3S. Full-profile Rietveld refinement of the irradiated $Ce_{0.58}Y_{0.15}Zr_{0.27}O_{1.93}$ – F1 sample in a Le Bail mode (R_P = 9.3%).

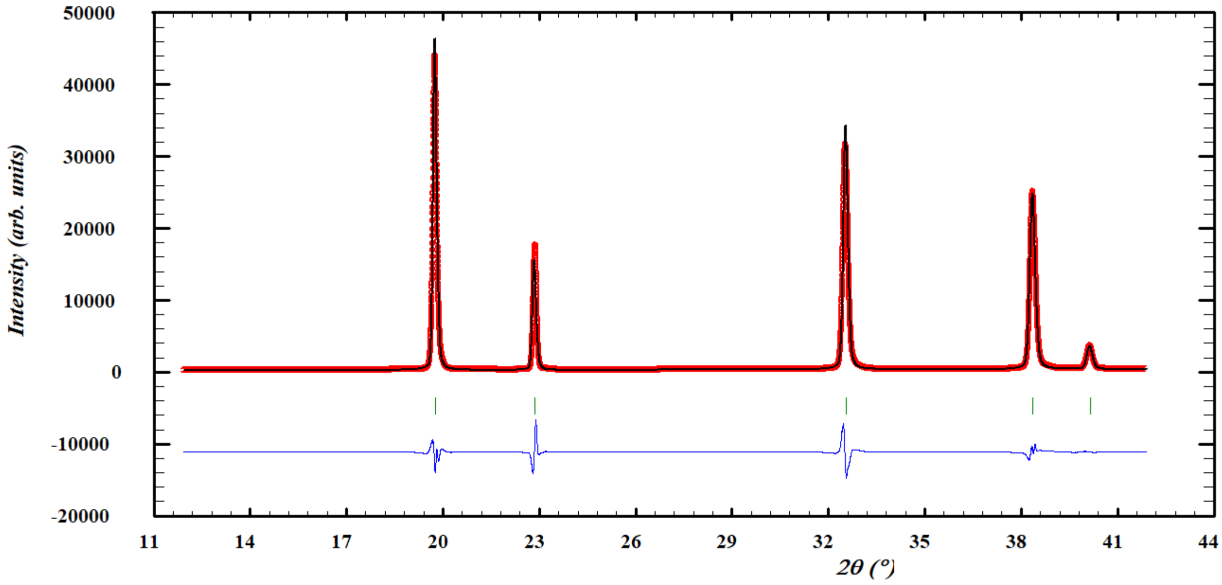

Fig. 4S. Full-profile Rietveld refinement of the irradiated $Ce_{0.18}Y_{0.15}Zr_{0.67}O_{1.93}$ – F2 sample in a Le Bail mode (R_P = 7.8%).

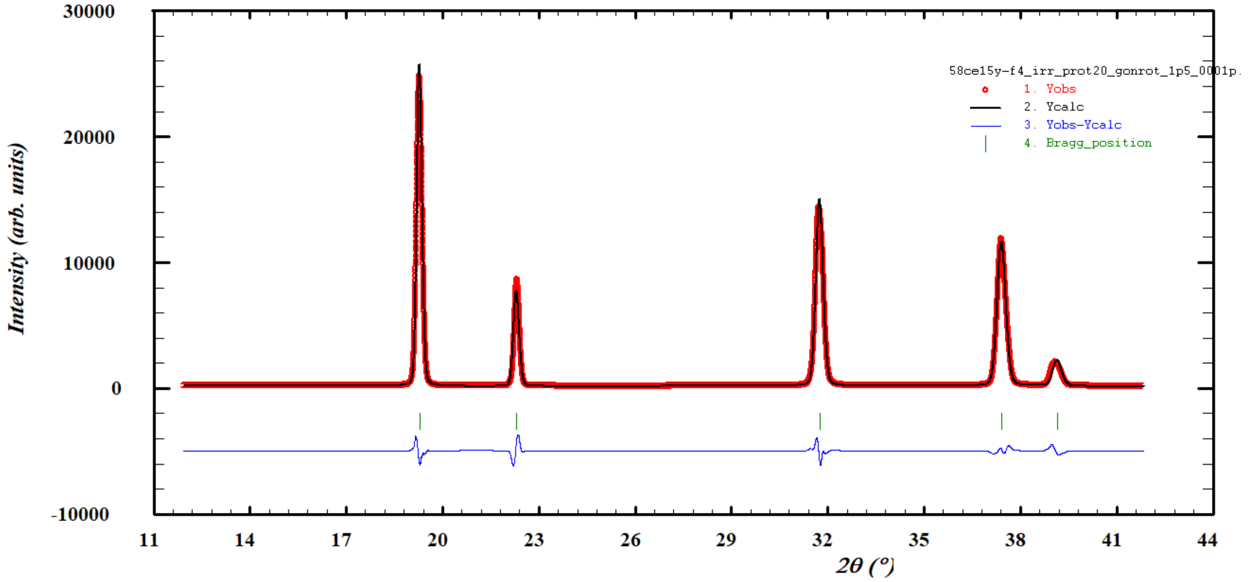

Fig. 5S. Full-profile Rietveld refinement of the irradiated $Ce_{0.58}Y_{0.15}Zr_{0.27}O_{1.93}$ – F2 sample in a Le Bail mode (R_P = 5.5%).

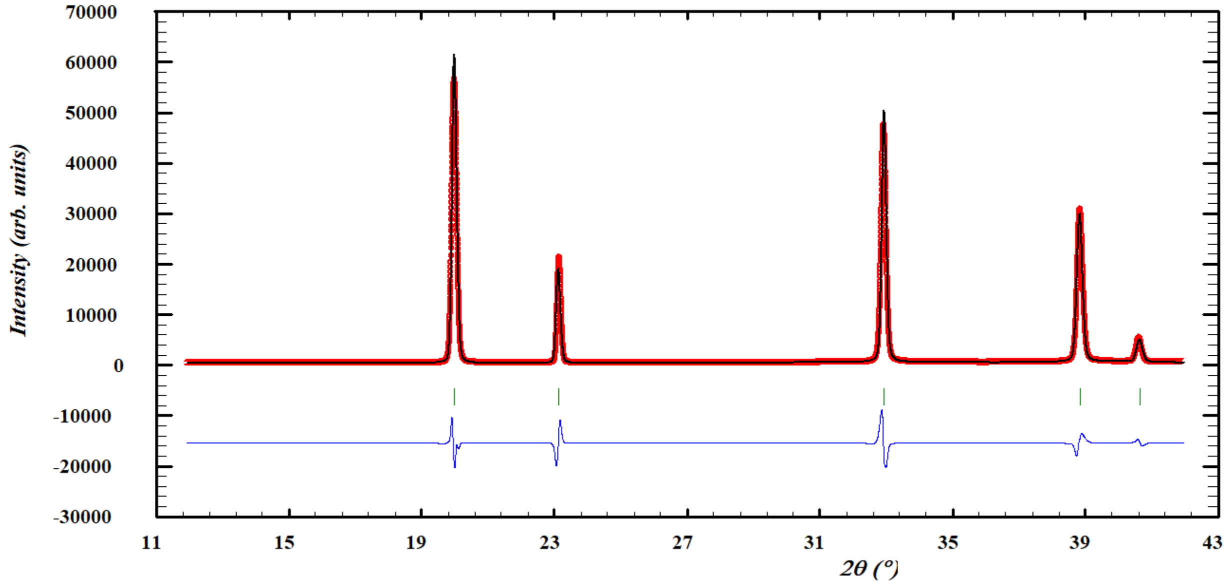

Fig. 6S. Full-profile Rietveld refinement of the irradiated YSZ – F2 sample in a Le Bail mode (R_P = 9.4%).

SRIM calculations illustrating penetration depth of 14 MeV Au ions into the Ce-doped YSZ and non-doped YSZ phases

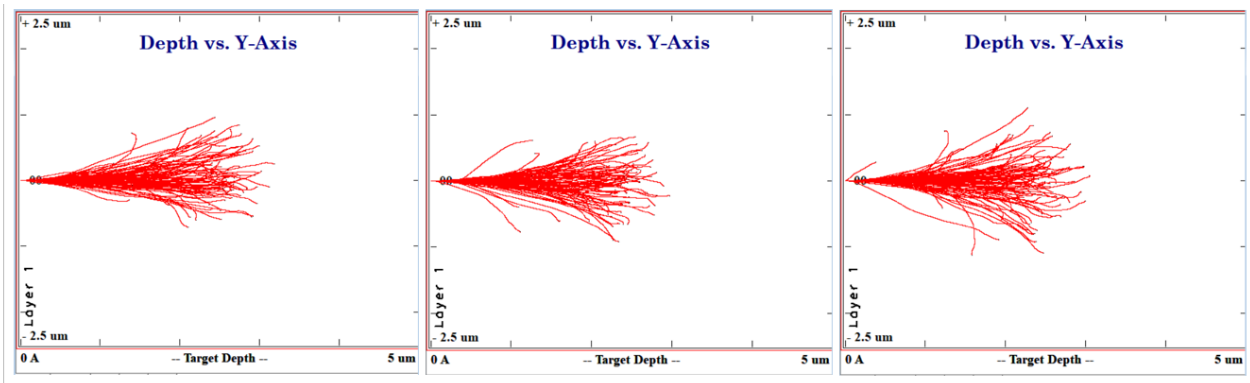

$Ce_{0.18}Y_{0.15}Zr_{0.67}O_{1.93}$ $Ce_{0.18}Y_{0.20}Zr_{0.62}O_{1.90}$ $Ce_{0.58}Y_{0.15}Zr_{0.27}O_{1.93}$

Fig. 7S. SRIM calculations illustrating penetration depth of 14 MeV Au ions into the Ce-doped YSZ phases: $Ce_{0.18}Y_{0.15}Zr_{0.67}O_{1.93}$ - left, $Ce_{0.18}Y_{0.20}Zr_{0.62}O_{1.90}$ - middle, and $Ce_{0.58}Y_{0.15}Zr_{0.27}O_{1.93}$ - right.

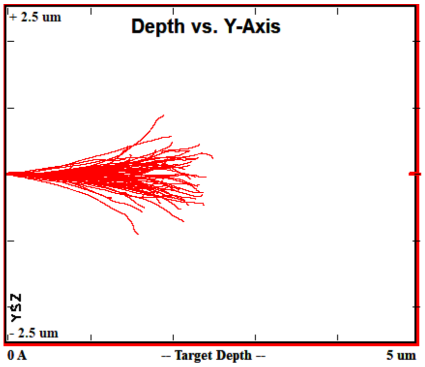

Fig. 8S. SRIM calculations illustrating penetration depth of 14 MeV Au ions into the YSZ ($Y_{0.15}Zr_{0.85}O_{1.93}$) phase
